# Does Casting Material Influence the Number of Casts Required Before Achilles Tenotomy in the Ponseti Treatment of Severe Idiopathic Clubfoot?

**DOI:** 10.3390/jcm15103924

**Published:** 2026-05-19

**Authors:** Valentina Di Carlo, Giulia Colin, Lucio Torelli, Michela Zorzi, Adamo Pio d’Adamo, Marco Carbone, Daniela Dibello

**Affiliations:** 1 Unit of Pediatric Orthopaedics and Traumatology, Institute for Maternal and Child Health—IRCCS “Burlo Garofolo”, 34137 Trieste, Italy; 2 Department of Medical, Surgical and Health Sciences University of Trieste, Strada di Fiume, 34129 Trieste, Italy; 3 Medical Genetics Unit, Institute for Maternal and Child Health—IRCCS “Burlo Garofolo”, 34137 Trieste, Italy; 4 Unit of Pediatric Orthopaedics and Traumatology, Giovanni XIII Children’s Hospital, Via Giovanni Amendola, 70126 Bari, Italy

**Keywords:** clubfoot, cast materials, Ponseti method

## Abstract

**Background**: Clubfoot represents a prevalent congenital deformity of the foot and ankle complex that may significantly compromise a child’s walking ability. Contemporary treatment protocols encompass serial manipulations and casting procedures designed to achieve gradual correction of the deformity. Various casting materials have been employed in this therapeutic approach, with plaster of Paris and fiberglass constituting the two predominant options. This study aimed to evaluate the comparative effectiveness of these casting materials and determine whether material selection influences the rate of correction and the clinical indications, specifically regarding the number of casts required before percutaneous Achilles tenotomy. **Methods**: We conducted a retrospective analysis of prospectively collected data on paediatric patients treated at our tertiary-level institution with both plaster of Paris (POP) and semirigid fiberglass (SRF) by a single orthopaedic surgeon between 2010 and 2020. Treatment was initiated within the first 30 days of life (median age 12 days, range 0–28 days). To reduce confounding bias related to baseline aetiology (e.g., rigid syndromic feet), the primary comparative analysis was restricted to the idiopathic clubfoot subgroup. The Pirani score was used to assess deformity severity at each clinical visit. **Results**: A cohort of 84 patients (137 feet) was enrolled and treated, comprising patients with a Pirani score ≥ 4.5, excluding non-idiopathic cases. The mean number of casts required was 5.8 ± 1.0 for POP and 5.7 ± 1.2 for SRF, with no statistically significant difference (*p* = 0.91). **Conclusions**: Both plaster of Paris and semirigid fiberglass are highly effective casting materials for the initial phase of Ponseti treatment. Both achieve comparable correction sufficient to proceed with Achilles tenotomy. Accordingly, material selection should be guided by clinician proficiency, institutional cost-effectiveness, and patient comfort. Further investigation is needed to evaluate long-term outcomes and the relative benefits of each material in clubfoot management.

## 1. Introduction

**Figure 1:**
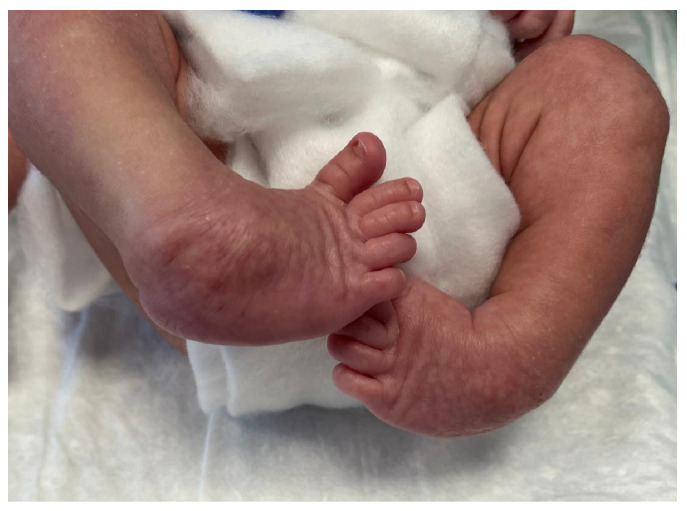
Clinical presentation of bilateral congenital talipes equinovarus (clubfoot) in a newborn, showing the characteristic hindfoot equinus, hindfoot varus, forefoot adductus, and midfoot cavus components of the deformity.

Clubfoot, also known as talipes equinovarus (TEV), represents a complex three-dimensional congenital deformity of the foot and ankle (Figure 1) with a global incidence of approximately 1 in every 1000 live births [1], and a regional rate of 1.09 per 1000 in northern Italy [2]. The Ponseti method is universally recognised as the gold standard treatment for clubfoot [3].

This non-surgical intervention encompasses a systematic series of gentle manipulations and sequential casting procedures designed to progressively correct the cavus, adductus, and varus components of the deformity through the biomechanical principles of creep and stress relaxation [4]. As a treatment modality specifically tailored for the neonatal and infant population, the Ponseti method achieves optimal results when initiated early in life. The enhanced tissue pliability during the neonatal period facilitates correction, with treatment ideally starting within the first weeks of life. After successful correction of the midfoot and forefoot, the residual equinus component is addressed by percutaneous Achilles tenotomy, followed by an orthotic bracing protocol to minimise the risk of recurrence [5,6].

The Ponseti method has demonstrated robust efficacy in achieving clubfoot correction, with the majority of patients attaining excellent functional outcomes and achieving normal walking and running capabilities [6]. In cases presenting with severe deformity or demonstrating resistance to conservative management, surgical intervention may be considered as a definitive treatment option.

Previous studies have reported high levels of parental satisfaction with the Ponseti method, with families perceiving it as an effective treatment despite the challenges of the casting phase. The quality of the physician–family relationship has been identified as a key determinant of treatment adherence and long-term outcomes [7].

An additional variable in the delivery of the Ponseti method is the choice of casting material. While plaster of Paris (POP) remains the most widely described material in the literature, semirigid fiberglass (SRF) has gained increasing clinical adoption in recent decades as an alternative with distinct mechanical and practical properties, due to its enhanced durability and flexibility [8,9]. These two materials differ in several clinically relevant respects.

POP has been used for decades in clubfoot treatment. Its superior mouldability makes it well suited for fabricating custom-fitted casts, and its low cost renders it accessible to most healthcare institutions. However, POP casts possess inherent limitations, including substantial weight and rigidity, which may compromise patient comfort. Additionally, they carry a greater risk of skin irritation in neonates during application and removal [9,10].

SRF, by contrast, offers reduced weight, radiolucency and waterproof properties, improving patient comfort and compliance, and is associated with a lower risk of thermal burns and a cleaner application process. Nevertheless, fiberglass casts entail greater material costs compared to plaster of Paris, which can be a drawback for some healthcare facilities. Furthermore, fiberglass casts can be more challenging to mould and shape compared to plaster of Paris casts [11,12].

At our tertiary-level institution, both casting materials are in use, with selection determined by individual clinician preference and the severity of deformity as assessed by the Pirani score.

While previous studies have focused primarily on parental satisfaction and mechanical properties [7,11], it remains unclear whether the biomechanical properties of different casting materials influence the rate of deformity correction.

Based on our clinical experience, we hypothesised that both materials represent viable therapeutic options for clubfoot treatment, given that all patients successfully progressed to the tenotomy phase uneventfully.

This study aimed to determine whether the choice of casting material—plaster of Paris or semirigid fiberglass—influences the number of casts required before Achilles tenotomy in infants with idiopathic clubfoot treated with the Ponseti method. The number of casts needed to reach the tenotomy threshold was used as the primary outcome, as it reflects both the severity and the correctability of the deformity and represents a practical, objective proxy for the rate of correction.

## 2. Materials and Methods

We conducted a retrospective analysis of prospectively collected data on paediatric patients treated for congenital clubfoot at our tertiary-level Maternal and Child Health Institution—IRCCS Burlo Garofolo, Trieste, Italy, using data collected between 2010 and 2020.

Upon presentation to our institution, patients were consecutively enrolled and assigned to receive treatment with either POP or SRF, based primarily on material availability and the clinical workflow at the time of the initial visit. To eliminate inter-operator variability and technical bias, all manipulations and casting procedures were performed by a single, highly experienced paediatric orthopaedic surgeon. Pirani score assessments at each visit were also performed by the same surgeon, ensuring consistency of outcome measurement; the potential for measurement bias inherent in this approach is acknowledged as a study limitation. Any episode of cast slip-out requiring unscheduled recasting was recorded in the clinical database; patients requiring premature cast replacement due to cast failure or slip-out were noted and the additional cast was counted in the total cast number for that patient.

Inclusion criteria were: (1) diagnosis of idiopathic congenital clubfoot; (2) treatment initiated within the first 30 days of life; and (3) an initial severity defined by a Pirani score strictly ≥ 4.5. Exclusion criteria comprised: (1) patients in whom the casting material was altered or mixed during the treatment course; (2) syndromic conditions, (3) an initial severity defined by a Pirani score < 4.5, (4) treatment started after the first 30 days of life, and (5) loss to follow-up prior to the tenotomy phase. The Pirani score threshold of ≥4.5 was chosen to restrict the analysis to severe clubfoot presentations, thereby ensuring a homogeneous cohort with sufficient deformity severity to require the full casting correction phase and subsequent Achilles tenotomy. The endpoint for tenotomy eligibility was defined as a Pirani score = 1, indicating satisfactory correction of the cavus, adductus, and varus components of the deformity.

All newborns were managed strictly according to the standardised Ponseti method and started treatment during the first month of life, with a median age of 12 days.

**Figure 2:**
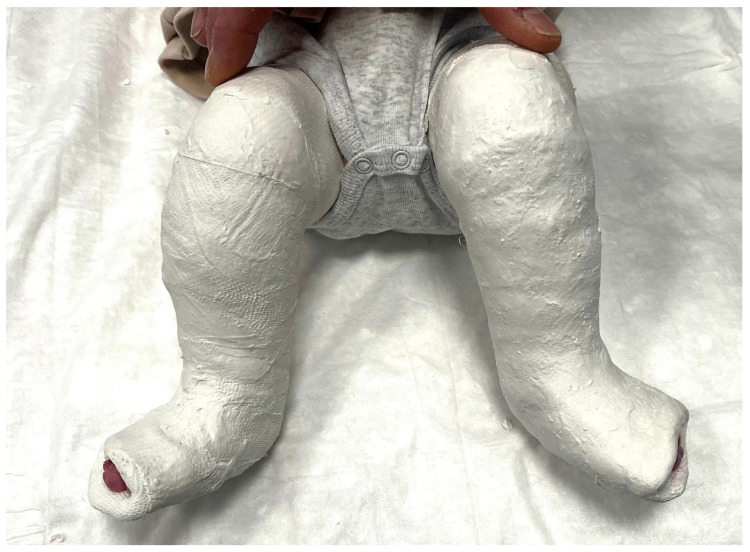
Plaster of Paris (POP) cast applied to an infant with bilateral clubfoot during the Ponseti serial casting protocol. The cast extends from the toes to the upper thigh with the knee flexed at 90°.

**Figure 3:**
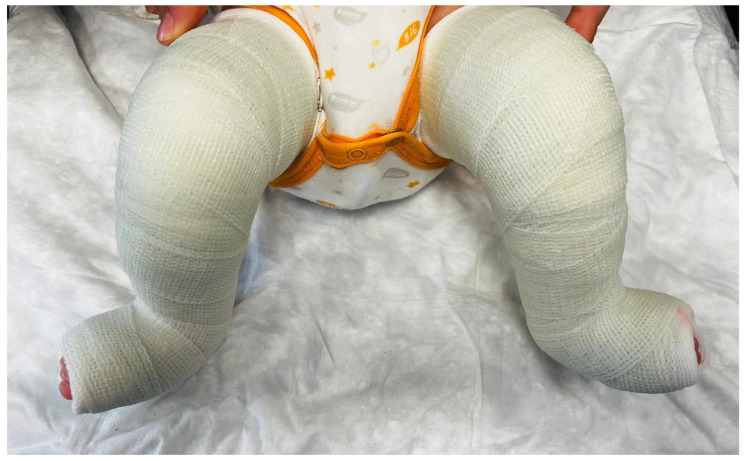
Semirigid fiberglass (SRF) cast applied to an infant with bilateral clubfoot during the Ponseti serial casting protocol. The casts extend from the toes to the upper thigh with the knee flexed at 90°.

Casts were applied from the toes to the upper proximal thigh, with the knee flexed at 90° (Figure 2 and Figure 3). Casts were removed, the foot gently manipulated, and a new cast applied on a weekly basis.

The Pirani scoring was employed to assess the severity of each foot prior to every cast application [13]. In cases of bilateral involvement, the number of casts employed for treatment was determined by the severity of the more affected foot, as indicated by the higher Pirani score at initial assessment. Once satisfactory correction of the cavus, adductus, and varus components had been achieved, patients were deemed suitable candidates for percutaneous Achilles tenotomy.

Demographic and clinical data were systematically collected in a dedicated database, encompassing patient sex, number of casts administered prior to tenotomy, laterality of involvement (unilateral vs. bilateral) and initial Pirani score.

Statistical analysis was performed using Python (SciPy version 1.11). Continuous variables were reported as mean ± standard deviation (SD) and range, while categorical variables were expressed as frequencies and percentages. The Shapiro–Wilk test was used to assess data normality. As data were not normally distributed (Shapiro–Wilk *p* < 0.01 for both groups), comparisons between the POP and SRF groups for the number of casts were conducted using the Mann–Whitney U test. Categorical variables were compared using Fisher’s exact test. A *p*-value < 0.05 was considered statistically significant.

The primary unit of analysis was the foot rather than the patient, in alignment with current clubfoot literature in which outcomes are conventionally reported per foot. This choice is clinically justified as each foot may present with a different degree of severity and may respond independently to treatment; in bilateral cases, each foot was therefore counted as a separate observation. No a priori sample size calculation was performed, as this study was designed as an exploratory observational study leveraging all available data collected over the 10-year study period. A post hoc power analysis yielded an estimated statistical power of approximately 60%, which is acknowledged as a limitation. To address the potential confounding effect of the sex imbalance between groups, a stratified Mann–Whitney U test was additionally performed separately for male and female patients.

## 3. Results

During the study period, a total of 107 patients were initially evaluated. Twenty-three patients were excluded due to a Pirani score < 4.5 or treatment started after the first 30 days of life. The final cohort consisted of 84 patients (137 feet) who completed the casting phase. The cohort included both male and female patients with Pirani scores exceeding 4.5 (range 4.5–6), encompassing both idiopathic and atypical presentations (e.g., stiff and complex foot). No patients were lost to follow-up during the casting phase.

The patients were divided into two groups based on the applied casting material: patients treated with POP totaled 46 (28 males and 18 females), of these 28 had bilateral TEV and 18 had monolateral TEV (8 right, 10 left). Patients treated with SRF totaled 38 (35 males and 3 females), of these 25 had bilateral TEV and 13 had monolateral TEV (7 right, 6 left). (Table 1).

All patients presented with idiopathic clubfoot (including atypical, complex and positional variants) and started treatment within the first 30 days of life (median age 12 days, range 0–28 days).

A significant imbalance was observed in sex distribution, with a higher proportion of males in the SRF group (92%) compared to the POP group (61%; Fisher’s exact test *p* = 0.001). However, sex is not an established predictor of response to Ponseti casting. Nevertheless, given the baseline imbalance between groups, a stratified Mann–Whitney U test was conducted separately for male and female patients; no significant difference in the number of casts required was observed in either subgroup, further supporting the validity of the primary outcome.

The average initial Pirani score was 5.8 ± 0.4 in the POP cohort (range 4.5–6), and 6.0 ± 0.2 for the SRF group (range 5.0–6).

**Figure 4:**
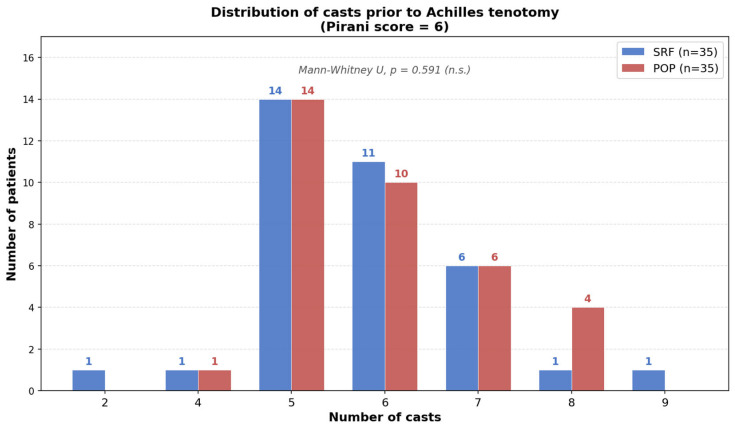
Distribution of the number of casts required prior to Achilles tenotomy in patients presenting with a Pirani score of 6, stratified by casting material: semirigid fiberglass (SRF, blue) versus plaster of Paris (POP, red). No statistically significant (n.s.) difference was observed between the two groups (*p* > 0.05).

The average number of casts in the SRF group was 5.7 ± 1.2 and for the POP group 5.8 ± 1.0 (Mann–Whitney U test, *p* = 0.91) with no significant difference. Considering only the idiopathic clubfoot subgroup (43 POP vs. 36 SRF patients), the average number of casts was 5.8 ± 1.0 for POP and 5.7 ± 1.2 for SRF (Mann–Whitney U test, *p* = 0.91). Restricting the analysis to patients with a Pirani score of 6, the average number of casts was 5.9 ± 1.1 for POP (*n* = 35) and 5.7 ± 1.2 for SRF (*n* = 35; Mann–Whitney U test, *p* = 0.59). Of the 84 patients, 5 had missing data on the number of casts applied (3 POP, 2 SRF) and were excluded from the cast count analysis, leaving 43 POP and 36 SRF patients available for comparison. None of these comparisons reached statistical significance (Table 2) (Figure 4).

Achilles tendon tenotomy was performed in 79 out of 84 patients (94%) at the end of the casting phase. The five patients (3 POP, 2 SRF) who did not undergo tenotomy had achieved satisfactory correction of the equinus component through casting alone, confirming that both materials support appropriate clinical progression through the treatment protocol. No major skin complications were recorded in either group during the casting phase. No cast saw injuries, thermal burns related to the exothermic setting reaction of POP, or significant pressure-related skin lesions were observed. Occasional minor reactions, such as transient erythema at pressure points, resolved spontaneously without clinical intervention and were not classified as adverse events.

Seventeen patients (10 POP, 7 SRF) experienced at least one episode of cast proximal migration; in all cases, the cast was promptly replaced and treatment continued without interruption, with no impact on the number of casts required to reach the tenotomy threshold.

## 4. Discussion

The primary objective of this retrospective analysis of prospectively collected data was to determine whether the intrinsic biomechanical properties of the casting material—traditional POP versus contemporary SRF—influence the speed of deformity correction during the conservative management of severe clubfoot using the Ponseti method.

This technique achieves gradual elongation of ligamentous and muscular structures through the biomechanical principles of creep and stress relaxation, exploiting the viscoelastic properties of the affected tissues [4]. The Ponseti method has transformed the management of clubfoot by substantially reducing the need for extensive surgical correction while improving long-term functional outcomes [14].

Theoretically, the superior rigidity, stress-relaxation properties, and unyielding moulding capability of POP could result in a more efficient transfer of corrective multi-planar forces to the dense ligamentous structures of the infant foot, potentially accelerating the correction rate. Indeed, POP remains the most frequently described material employed for this therapeutic purpose within the published literature [8]. However, our study findings for patients presenting with severe clubfoot (Pirani score > 4.5) do not substantiate this theoretical premise.

Specifically, when restricting the analysis to idiopathic clubfoot to exclude the confounding rigidity of syndromic cases, the mean number of casts was similar between the two groups: 5.8 ± 1.0 for POP compared to 5.7 ± 1.2 for SRF (43 POP vs. 36 SRF patients), with no statistically significant difference (Mann–Whitney U test, *p* = 0.91). These findings suggest that, when the Ponseti technique is properly applied, the choice of casting material does not significantly influence the rate of deformity correction.

Stratified analysis restricted to patients with a Pirani score of 6 yielded consistent results: the mean number of casts remained comparable between materials (POP: 5.9 ± 1.1 vs. SRF: 5.7 ± 1.2; *p* = 0.59), further confirming the absence of a material effect on correction rate (Table 2).

Our results contextualize and expand upon the ongoing scholarly debate regarding material optimization. Previous comparative trials have yielded mixed results: Pittner et al. conducted a prospective randomised trial demonstrating superior final severity scores for the POP group (4.2) relative to the SRF group (6.4). Conversely, the investigators reported enhanced overall material satisfaction metrics for fiberglass, encompassing cast convenience and parental willingness to recommend the material to others [8]. These findings align with those reported by Coss et al., who demonstrated statistical superiority of fiberglass with respect to durability, waterproofing, performance characteristics, and ease of removal despite POP’s superior initial molding properties [11]. While plaster demonstrated superior severity scores, fiberglass was favoured for its enhanced material satisfaction profile and practical advantages.

A more recent comparative analysis by Monforte et al. examined the use of POP versus SRF in idiopathic clubfoot treated with the Ponseti method, finding that SRF—despite its slightly reduced moldability—achieves correction and recurrence rates comparable to POP when used by experienced practitioners. In conclusion, both materials exhibited similar efficacy regarding correction achievement and recurrence rates [15].

Our data strongly align with this modern consensus: in expert hands, the “memory” effect of fiberglass does not impede the progressive correction required to achieve 60 degrees of abduction and meet the clinical indication for Achilles tenotomy.

Beyond clinical efficacy, healthcare systems must consider resource allocation. At our institution, Cellona plaster of Paris rolls are available in three sizes: small (6 cm × 2 m) at €0.63, medium (10 cm × 2 m) at €0.94, and large (15 cm × 2 m) at €1.27. SRF rolls are considerably more expensive: small size (2.5 cm × 1.8 m) at €4.95 and medium size (5 cm × 3.6 m) at €6.43. Given the comparable average number of casts per patient (approximately 6), a full pre-tenotomy treatment course with SRF incurs substantially higher material costs than POP. However, this direct cost difference must be weighed against the operational advantages of SRF: shorter cast removal time, less ambient dust, greater resistance to urinary soiling, and higher parental satisfaction scores, as reported in previous comparative studies [8,11]. A comprehensive cost-effectiveness analysis should account for both material costs and these broader operational and experiential factors. It should be noted that the above represents a simple direct cost comparison rather than a formal cost-effectiveness analysis, as indirect costs such as application time, cast removal time, staff time, and complication management were not systematically recorded.

It is also important to acknowledge the significant baseline imbalances observed between the two groups. The POP group had a higher proportion of females (39% vs. 8%; *p* = 0.001). This marked sex imbalance is attributable to the observational nature of the study.

No major skin complications were recorded in either group. No cast saw injuries, thermal burns from the exothermic reaction of POP, or significant pressure-related skin lesions were observed. Occasional minor reactions, such as transient erythema at pressure points, resolved spontaneously without intervention and were not classified as adverse events. Both materials can therefore be considered safe for use in neonates when applied by an experienced operator.

Regarding cast stability, slippage was recorded in 17 patients. Among patients with bilateral clubfoot (*n* = 10), slippage occurred in only one foot per patient (5 feet total); the remaining 7 patients had unilateral involvement, yielding 12 episodes out of 137 treated feet (9%). None of these episodes affected overall treatment progression or the final number of casts required to reach the tenotomy threshold, confirming that occasional cast slippage does not compromise treatment efficacy when managed promptly.

## 5. Limitations

This study presents certain limitations that must be acknowledged. First, the lack of randomisation and the allocation of casting material based on availability and clinical workflow represent important methodological constraints that may have introduced selection bias, as reflected in the baseline sex imbalance between groups. While a stratified analysis confirmed that this imbalance did not affect the primary outcome, a randomised controlled design would be required to eliminate this potential confounding more rigorously. Second, blinding of the treating surgeon to the casting material was not feasible, introducing an inherent performance bias. Third, follow-up in this analysis was intentionally truncated at the time of tenotomy; therefore, we cannot draw conclusions regarding whether the initial casting material influences long-term outcomes, such as relapse rate at walking age. Fourth, as this was an exploratory observational study based on all available data from a ten-year period, no a priori sample size calculation was performed; post hoc power analysis estimated a statistical power of approximately 60%, which should be considered when interpreting the results. Finally, parental satisfaction, anxiety, and the psychosocial impact of caring for an infant in POP versus SRF were not quantified using validated Patient-Reported Outcome Measures (PROMs) [7].

## 6. Conclusions

In conclusion, both plaster of Paris and semirigid fiberglass constitute effective and safe options for the initial casting phase of the Ponseti method. While each material carries distinct practical advantages and limitations, neither confers a measurable benefit in terms of deformity correction rate, as evidenced by the comparable number of casts required before tenotomy in this cohort. Material selection should therefore be guided by clinician proficiency, patient comfort, and institutional cost considerations, with the shared goal of achieving optimal correction and maintaining adherence to established treatment protocols. Further studies incorporating validated parental satisfaction measures and long-term follow-up are warranted to fully characterise the comparative benefits of each material across the complete course of clubfoot management.

## Figures and Tables

**Table 1:** Baseline demographic and clinical characteristics of the study cohort.

Characteristic	Total Cohort (*n* = 84)	POP Group (*n* = 46)	SRF Group (*n* = 38)
Sex, *n* (%)			
-Male	63 (75.0%)	28 (60.9%)	35 (92.1%)
-Female	21 (25.0%)	18 (39.1%)	3 (7.9%)
Laterality, *n* (%)			
-Bilateral	53 (63.1%)	28 (60.9%)	25 (65.8%)
-Unilateral	31 (36.9%)	18 (39.1%)	13 (34.2%)
Initial Pirani Score	5.89 ± 0.33	5.83 ± 0.41	5.96 ± 0.18

Categorical variables compared with Fisher’s exact test; continuous variables compared with Mann–Whitney U test.

**Table 2:** Number of casts required prior to Achilles tenotomy, stratified by casting material and subgroup analysis.

Analysis	POP (Mean ± SD)	SRF (Mean ± SD)	U Statistic
Idiopathic only (*n* = 43 vs. 36)	5.81 ± 1.03	5.72 ± 1.21	785.5
Pirani = 6 only (*n* = 35 vs. 35)	5.94 ± 1.08	5.74 ± 1.22	656.5

Comparisons performed using the Mann–Whitney U test (two-sided). SD = standard deviation.

## Data Availability

The data underlying this study are available upon request from the corresponding author.

## Abbreviations

The following abbreviations are used in this manuscript:

TEV	Talipes equinovarus
POP	Plaster of Paris
SRF	Semirigid fiberglass
